# Prevalence and clinical factors associated with survival in patients with EGFR-mutated lung cancer in Argentina

**DOI:** 10.3332/ecancer.2024.1737

**Published:** 2024-08-14

**Authors:** Luis Basbus, Sergio Specterman, Lorena Lupinacci, Federico Cayol

**Affiliations:** Clinical Oncology Section, Hospital Italiano de Buenos Aires, Buenos Aires 1199, Argentina

**Keywords:** lung cancer, epidermal growth factor receptor, tyrosine kinase inhibitors

## Abstract

**Introduction:**

Lung cancer remains a leading cause of cancer-related mortality worldwide. Detecting mutations in the epidermal growth factor receptor (EGFR) is crucial for treatment selection due to the response to tyrosine kinase inhibitors (TKIs) in these patients.

**Objective:**

Describe the prevalence and identify factors associated with survival in stage IV lung cancer patients harboring EGFR mutations in a real-world setting.

**Materials and methods:**

A retrospective cohort study was conducted to identify factors associated with progression-free survival (PFS), overall survival (OS) and response rate in stage IV lung cancer patients with EGFR mutations.

**Results:**

Data from 771 patients diagnosed with lung cancer between 2017 and 2021 at the Hospital Italiano de Buenos Aires were analysed. The prevalence of EGFR mutations was 18% (139), with a median follow-up of 30 months. Of these, 118 were treated with EGFR TKIs, with a higher objective response rate observed with osimertinib compared to first or second-generation TKIs. Adverse prognostic factors included an ECOG performance status greater than 1, uncommon mutations, high disease burden and the presence of brain or hepatic metastases. Osimertinib was associated with a reduced risk of progression or death, even after adjusting for these prognostic factors. The median PFS was 13 months, with a significant OS difference between patients treated with osimertinib versus first or second-generation inhibitors.

**Conclusion:**

This study underscores the importance of EGFR mutation detection in stage IV lung cancer patients and supports the need for personalised therapeutic approaches to improve outcomes in this patient population.

## Introduction

Lung cancer is the leading cause of cancer-related mortality worldwide [1]. Approximately 40% of patients are diagnosed at advanced stages and thus require systemic treatment [2]. Currently, there are several possible treatments available, such as chemotherapy, immunotherapy, tyrosine kinase inhibitors (TKIs) or their combination [3, 4].

The choice of treatment depends, among other parameters, on the presence of genetic alterations, such as mutations in the epidermal growth factor receptor (EGFR), a prognostic marker and predictive of response to TKIs, which varies among different series, approximately representing 15% of metastatic non-small cell lung cancers (mNSCLCs) [5]. In studies of Asian patients, such as the PIONEER study, the prevalence was much higher, with 51% of 1,450 patients presenting EGFR mutations [6]. Other parameters to evaluate include anaplastic lymphoma kinase and ROS1 kinase translocations [7, 8], or expression of programmed cell death receptor ligand-1 in the tumor [9].

Tumor growth and progression depend on the activity of cell surface receptors that control intracellular signaling pathways regulating proliferation, apoptosis, angiogenesis, adhesion and cellular motility [10].

These cell surface receptors include tyrosine kinases (TKs) of EGFR. EGFR exists as a monomer on the cell surface and must dimerize to activate the TK domain. While EGFR TK activity is tightly controlled in normal cells, genes encoding these receptors may have escaped their usual intracellular inhibitory mechanisms in malignant cells [11].

In Argentina, there are few published data; in a series of 110 patients analysed at CEMIC, 11 had EGFR mutation [12]. In a multicenter series from our country recently published of 431 stage IV lung cancer patients, 79% were tested for EGFR, finding a prevalence of EGFR of 22% [13].

Currently, the treatment of patients diagnosed with lung cancer who present sensitive EGFR mutations is basically defined by the result of mutation detection in tumor tissue biopsy or liquid biopsy in blood. Its presence, whether a frequent or infrequent mutation, indicates the initiation of treatment with TKIs, either first, second, or third generation, as these significantly improve outcomes compared to platinum-based chemotherapy doublets [14]. In the context of limited access, as in countries like Argentina, a commonly employed strategy involves the sequential administration of treatments, commencing with first- or second-generation TKIs. [15].

Although first-generation (erlotinib and gefitinib) and second-generation (afatinib and dacomatinib) TKIs have been the standard treatment for initial EGFR treatment, the data suggest better outcomes with first-line treatment with the third-generation agent, osimertinib [16].

In the phase III FLAURA trial, 556 untreated advanced NSCLC patients with EGFR mutation were randomly assigned to osimertinib versus standard of care (SOC) EGFR TKI (gefitinib or erlotinib). Osimertinib demonstrated an improvement in progression-free survival (PFS) of 18.9 versus 10.2 months [17].

In the report of overall survival (OS) results at 58% maturity, osimertinib improved OS compared to SOC (38.6 versus 31.8 months). Response rates for osimertinib and SOC were 80% and 76%, respectively [18].

However, as with other types of solid tumors where the success of driver treatments has been demonstrated with TKI use, eventually, the disease progresses. The reported median in mutated EGFR NSCLC is around 40 months [19]. Thus, seeking prognostic factors and underlying resistance mechanisms is crucial to improving treatment choices.

In case of difficulty in accessing third-generation TKIs, the use of first or second-generation TKIs should be integrated with other treatment strategies to prolong survival and maintain quality of life for as long as possible. Patients progressing on a first or second-generation TKI show sensitivity and good outcomes to treatment with osimertinib when diagnosed with a threonine to methionine substitution at position 790 (T790M) as the resistance mechanism [20].

For patients without a T790M mutation, or those progressing on osimertinib, combined chemotherapy is usually the next treatment option with relatively short survivals in our country [18].

## Objectives

### Primary objectives

- Estimate the prevalence of positive EGFR mutation tests in the population. Analyse the type of mutation as well as the prevalence of uncommon mutations.
- Identify factors associated with increased PFS, OS and response rate to EGFR TKIs in patients with mNSCLC harboring EGFR mutations.

### Secondary objectives

- Identify factors associated with primary resistance (disease progression despite treatment) to these inhibitors.
- Describe the OS of patients with NSCLC harboring EGFR mutation.
- Evaluate clinical characteristics associated with higher response rates to treatment with EGFR inhibitors.
- Estimate the association of mutation type (common or uncommon) present in the tissue with patients' baseline characteristics.
- Analyse the prevalence of progression through T790M mutation and the median PFS2 in patients progressing through this mechanism.

## Materials and methods

An observational retrospective cohort study was conducted on 771 patients diagnosed with mNSCLC at an Italian hospital between 2017 and 2021. All patients diagnosed with lung cancer eligible for oncology-specific treatment were tested for EGFR during the study period. Those who underwent EGFR testing were analysed to calculate the prevalence of positive tests. The baseline clinical characteristics of the patients were examined, with cohort entry defined as the date of pathological diagnosis. All patients had at least 6 months of follow-up with imaging evaluation.

Given the low frequency of the condition, all patients who met the defined criteria during the retrospective study period were included.

To detect a difference between the first and second-generation TKI groups compared to Osimertinib, assuming an anticipated hazard ratio (HR) of 0.5 based on prior studies, we aimed to observe a total of 66 progression events. With 104 patients experiencing progression, the study achieved a statistical power of 95%.

The Italian Hospital of Buenos Aires (HIBA) is a private university hospital of high complexity located in the Autonomous City of Buenos Aires. It has a unique repository of information for each patient centralised through an electronic health record, in which patients have voluntarily and certifiedly signed informed consent for the use of their data for research purposes. All study data were treated with maximum confidentiality and anonymously. The protocol was evaluated and approved by the Research Protocol Ethics Committee.

Selection criteria of inclusion:

- Age 18 years or older at diagnosis
- Diagnosis of lung cancer with histological biopsy
- Metastatic non-small cell variant with EGFR testing
- Patients who underwent treatment with EGFR TKIs were included if they had a follow-up period of at least 6 months, or if shorter, had died before reaching 6 months, and underwent reevaluation by oncology.

Regarding descriptive statistics, continuous quantitative variables were presented using mean and SD or median and interquartile range (IQR) according to distribution. Categorical variables were presented using proportions with their respective 95% confidence intervals (CIs).

### For analytical statistics, the following approaches were used

Univariate and bivariate modeling of baseline characteristics using the Cox proportional hazards model for those that were statistically significant for each survival and response model.

Survival analysis or time-to-event analysis for OS. Kaplan-Meier curves were used to select the sample according to responders and non-responders, with an estimation of Log rank test and presentation of estimated HR with their 95% CI and *p*-values of Wald tests for each potential predictor including baseline characteristics, followed by adjustment for possible confounders through a Cox regression model.

Probabilities less than 0.05 were considered statistically significant. Statistical analysis was performed using Stata statistical software, version 15.1.

### Ethical aspects

All study data were treated with maximum confidentiality, with restricted access only for authorised personnel for study purposes in accordance with current legal regulations National Law on Protection of Personal Data 25.326/00 (Habeas Data Law) and Law 26. 529 /09.

In total agreement with current national and international regulations: the Declaration of Ethical Principles for Medical Research Involving Human Subjects of the World Medical Association and subsequent amendments, Good Clinical Practice guidelines of the International Conference on Harmonisation ICH and local regulatory laws ANMAT Resolution No. 5330/97 (with modifications of ANMAT Resolution No. 690/2005, 1067/2008 and 6550/2008) and ANMAT Resolution No. 1310/09.

## Results

A total of 771 EGFR tests were conducted on stage IV lung cancer patients, diagnosing 141 patients with EGFR mutation during the period 2017–2021, with a prevalence of 18%. Among those, 118 patients met all inclusion criteria and none of the exclusion criteria. The main reasons for patient exclusion were either not receiving TKI treatment or lack of follow-up at HIBA. The median follow-up period was 30 months.

The median age was 69 years, with an IQR of 59–78 years, a higher prevalence of women at 64% (76), and a low prevalence of smoking in 34% (40) of patients. Regarding ECOG, 75% (88) had PS 0-1, while 17% (20) had ECOG 3-4.

Ninety-five percent (112) of the histologies corresponded to adenocarcinomas, while 5% (6) were squamous cell tumors. Regarding the type of EGFR mutation, 46% (54) corresponded to exon 19, followed by 41% (48) to exon 21, and 8% (10) of patients presented with an uncommon EGFR mutation.

The prevalence of brain metastases at diagnosis was 24% (28), and 13% (15) had hepatic metastases, with 28% (33) presenting with a high disease burden, defined as having more than 3 metastatic sites. Further demographic results are detailed in Table 1.

**Figure 1.**
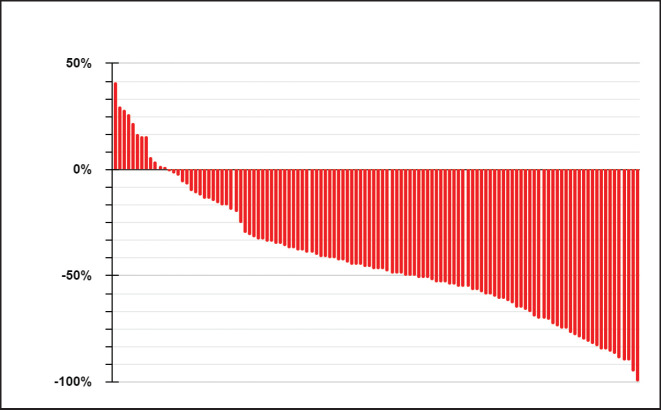
Response to TKIs according to RECIST 1.1.

Regarding treatment, 81% (95) initiated treatment with first- or second-generation TKIs, while 19% did so with a third-generation TKI, resulting in an objective response rate of 75%, with 7% (8) of patients experiencing primary progression to TKI (Figure 1).

A higher objective response rate was observed with third-generation TKIs compared to first- or second-generation ones, 91% versus 72% (χ^2^ *p* = 0.049). Of the ten patients with uncommon EGFR mutations, nine received first- or second-generation TKIs, and one patient received osimertinib, with a response rate of 40%.

Negative factors associated with not responding to TKIs in multivariate analysis were high disease burden, defined as three or more metastatic sites, with an OR of 4 (95% CI 1.4–11.2), ECOG greater than 1, OR 3 (95% CI 1.02–8.75) and uncommon mutation with OR 4.9 (95% CI 1.13–21).

Eighty-eight percent (104) experienced progression to TKI during follow-up, with a median time to progression or death (PFS) of 13 months, showing a difference in median PFS between patients receiving third-generation TKIs versus first- or second-generation ones, of 16 versus 12 months, by a Cox regression model with an HR of 0.51 (0.29–0.87) *p* = 0.008.

Of the 95 patients receiving first- or second-generation TKIs, 26% (25) progressed through T790M, of whom 24 patients received a third-generation TKI at progression, showing a PFS2 with osimertinib of 21 months (95% CI 8–29).

A Cox regression model was conducted to assess factors associated with lower PFS, revealing an association between having an ECOG less than 1, with an HR of 3.5 (95% CI 2.2–5.5), high disease burden, HR of 1.75 (95% CI 1.1–1.7), hepatic metastases HR 2 (95% CI 1.16–3.6), brain metastases and HR of 1.25 not statistically significant (95% CI 0.8–1.9).

The median OS for the entire cohort was 30 months, with a median in the osimertinib group of 34 months, compared to 29 months in the first- or second-generation TKI group, Cox regression HR 0.67 (95% CI 0.34–0.93).

A multivariate analysis was conducted, comparing the probability of progression or death with a third-generation TKI versus first- or second-generation ones, adjusting for the four variables that were statistically significant. Even after adjusting for these potential confounders, a 44% decrease in the risk of progression or death with a third-generation TKI persisted, with a Cox regression HR of 0.56 (95% CI 0.32–0.98).

## Discussion

This study provides valuable insights into the prevalence of EGFR mutation in our setting, which is similar to those reported in the literature for Latin American and European series, as well as clinical characteristics, treatment and outcomes in stage IV lung cancer patients.

Similar to other reported cohorts, patients exhibited specific demographic and clinical characteristics, such as a higher prevalence in women, a low proportion of smokers and an unequal distribution in terms of functional status.

Consistent with other studies, most patients had adenocarcinoma histologies, with the most common mutations found in EGFR exons 19 and 21.

The median PFS in the entire cohort was 13 months, with a significant difference in survival between patients treated with third-generation TKIs for 16 months and those treated with first- or second-generation TKIs for 12 months.

In the Cox regression analysis, several prognostic factors associated with lower PFS were identified, such as an ECOG functional status greater than 1, high disease burden, and the presence of brain and hepatic metastases. It was observed that the use of third-generation TKIs was associated with a lower probability of progression or death in the multivariate analysis, even after adjusting for these prognostic factors.

The treatment effectiveness findings are similar to phase 3 studies; however, being a real-life study with a notable proportion of patients with PS 3–4, the OS results are numerically lower than those reported in the FLAURA trial.

In patients who progressed to first- or second-generation TKIs, our cohort has a lower proportion of progression due to T790M mutation, with only 25% progressing through this mechanism, compared to other series reporting a prevalence of 50% in this scenario.

It is important in patients who do not have access to third-generation TKIs to search for this resistance mutation given the PFS2 of 21 months (95% CI 8–29).

Compared to the AURA study, in which the PFS was 10.1 months, this study reports a longer PFS. However, it should be noted that most patients evaluated for the T790M mutation correspond to long responders to first- or second-generation TKIs, representing a selection bias compared to the pivotal study. Additionally, with a small number of patients, the CI includes the 10.1 months reported in the AURA study.

## Conclusion

Given these results, we emphasize the importance of conducting EGFR mutation detection tests due to their high prevalence in our setting, which was 18% in our cohort.

It is important to consider functional status by ECOG, disease burden and central nervous system involvement as poor prognostic factors.

In terms of treatment, osimertinib showed a higher objective response rate compared to first- or second-generation TKIs, 91% versus 72%. Despite the high response rate of TKIs, a high progression rate was observed during follow-up, indicating the need to explore additional therapeutic strategies to improve outcomes in these patients, such as combining them with chemotherapy, a strategy recently published in the FLAURA2 study, as well as new treatment agents at progression, such as the combination of Amivantamab + Lazertinib.

When planning treatment, especially in the context of difficulty accessing third-generation inhibitors, their indication should be prioritised in populations at higher risk of progression or death.

In summary, these findings highlight the importance of detecting EGFR mutation in stage IV lung cancer patients and support the need for a personalised therapeutic approach. The appropriate choice of treatment can improve outcomes in terms of response and survival in this patient population. However, further research is needed to better understand resistance mechanisms and develop more effective therapeutic strategies.

The weaknesses of this study mainly correspond to its retrospective nature, which is exposed to multiple biases. Additionally, it is a single-center study characterised by the high volume of patients with this pathology.

## Conflicts of interest

The authors declare no conflicts of interest.

## Funding

This research did not receive any specific grant from funding agencies in the public, commercial or not-for-profit sectors.

## Figures and Tables

**Table 1.** Baseline characteristics of the population.

Clinical characteristics	*n* = 118
Age median (IQR)	69 (59–78)
Female gender *n* (%)	76 (64%)
Smoking *n* (%)	40 (34%)
ECOG 0-1 *n* (%)	88 (75%)
Histology: *n* (%)	
Adenocarcinoma	112 (95%)
Squamous	6 (5%)
Metastatic disease *n* (%)	
Brain	28 (24%)
Hepatic	15 (13%)
> 3 Territories	33 (28%)
